# Supplementary material for: Experiences of tobacco cessation including a prescription approach among patients in Swedish primary health care with a focus on socioeconomically disadvantaged areas
Source: PLoS One. 2020 Oct 12;15(10):e0240411. doi: 10.1371/journal.pone.0240411 (PMC7549804; doi:10.1371/journal.pone.0240411)
Supplement: S1 Appendix — (DOCX) [file pone.0240411.s001.docx]

# **S1 Appendix. Interview guide.**

## **Experiences of trying to quit tobacco use**

1. What are your experiences of trying to quit tobacco use?
2. How many times have you tried to quit your tobacco use?
   1. When?
   2. Why?
3. How did you go about it when you tried to quit your tobacco use?
   1. What was difficult/which barriers did you experience when you tried to quit your tobacco use?
   2. What helped you/made it easier when you tried to quit your tobacco use?

## **Support from the primary healthcare center**

1. What support did you receive from your primary healthcare center to quit your tobacco use?
   1. From who?
   2. When was this?
   3. How did they support you?
   4. What support was missing?
2. How did you perceive the support that you received from your primary healthcare center to quit your tobacco use?
   1. What worked well? In what way?
   2. What could have worked better? In what way?

## **Tobacco Cessation on Prescription**

1. Do you recognize this form [Tobacco Cessation on Prescription]?
   1. If yes, from where? How was it used with the counseling at the primary healthcare center? How did you perceive the form [Tobacco Cessation on Prescription]?
   2. If no, how do you perceive the form [Tobacco Cessation on Prescription] now when you have it in front of you?
   3. What was positive about it? In what way?
   4. What was negative about it? In what way?

## **Other questions**

1. How do you perceive your tobacco use today? Why?
   1. What support would you like to have in the future to quit your tobacco use?
2. What advice would you give to a friend who wants to quit their tobacco use?
3. What support, in addition to the support you have received from your primary healthcare center, could make it easier for you to quit your tobacco use?

## **Ending**

1. What else would you like to add or tell that could be important to know about your experiences of trying to quit your tobacco use?
2. What else would you like to add or tell that could be important to know about the support you have received from the primary healthcare center?
3. How did you perceive this interview?
