# Supplementary figures and images for: Experiences of tobacco cessation including a prescription approach among patients in Swedish primary health care with a focus on socioeconomically disadvantaged areas
Source: PLoS One. 2020 Oct 12;15(10):e0240411. doi: 10.1371/journal.pone.0240411 (PMC7549804; doi:10.1371/journal.pone.0240411)

# **S2 Appendix. Prescription form.**


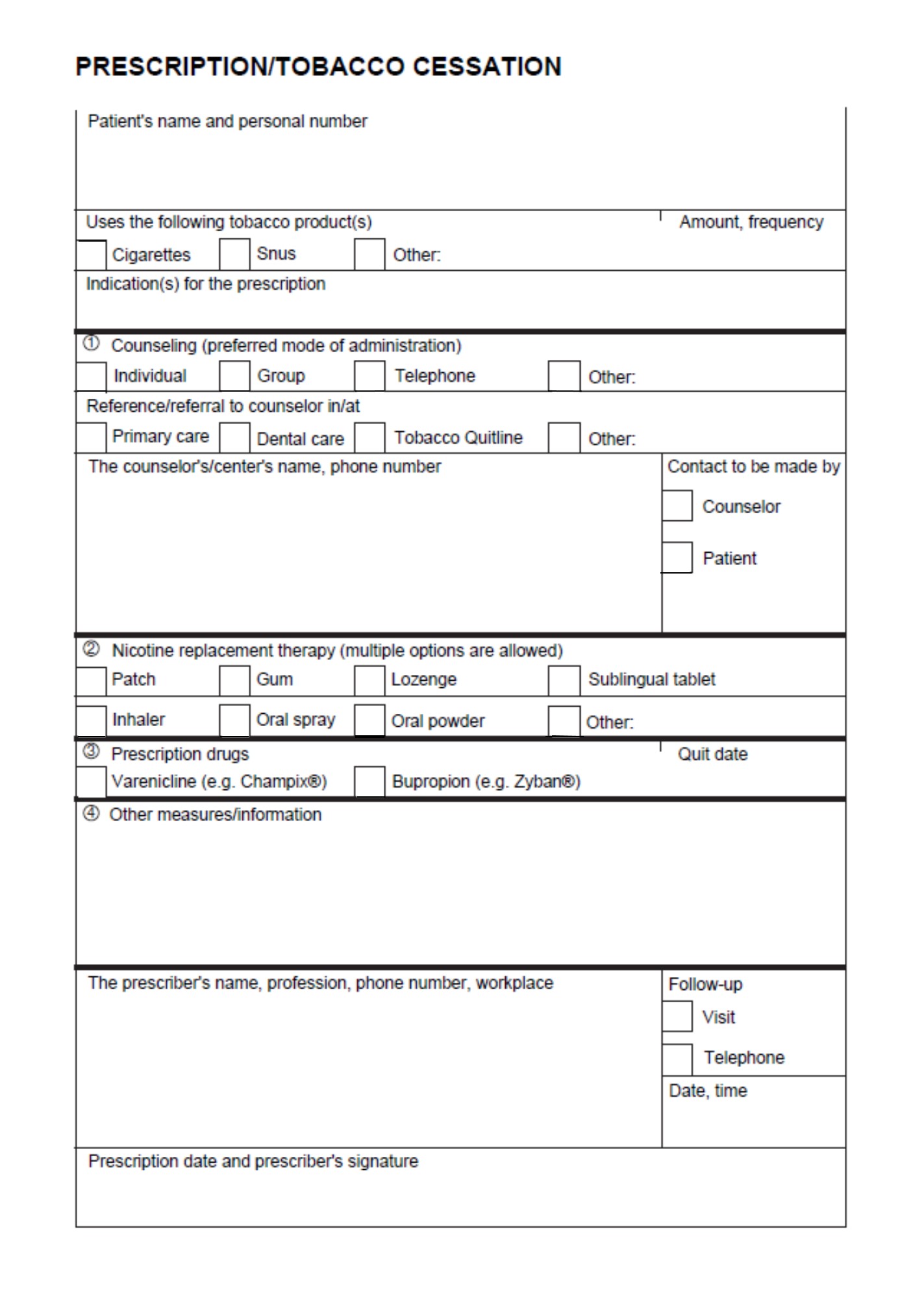


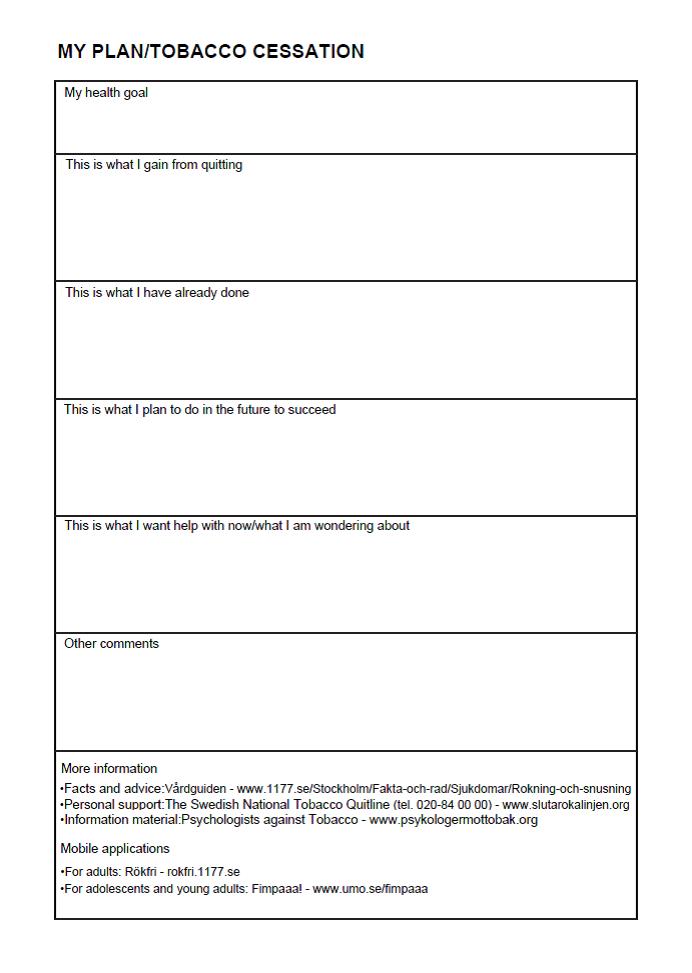

Supplement: S2 Appendix — (DOCX) [file pone.0240411.s002.docx]
